# Enhancing pragmatic competence in Kurdish EFL learners: The impact of a learner-centered approach

**DOI:** 10.1371/journal.pone.0334857

**Published:** 2025-11-07

**Authors:** Zhikal Qader Salih, Mustafa Kurt

**Affiliations:** 1 Department of English Language Education, Near East University, Nicosia, North Cyprus; 2 Department of English Language Teaching, Near East University, Nicosia, North Cyprus; Universiti Sains Malaysia, MALAYSIA

## Abstract

The study investigates how a learner-centered learning approach (LCL) can help raise pragmatic competence in Kurdish learners of English as a foreign language (EFL). Pragmatic competence in using languages appropriately in societal contexts is not well-developed in Kurdish learners because of traditional grammar-based teaching practices. The study attempted to investigate how LCL could foster effective usage of English in natural communication contexts. Survey employed a quantitative research design using an adapted Discourse Completion Test (DCT) and formal questionnaires administered to 98 EFL learners and 12 teachers. Learners were taught LCL-based methodology for a defined period and included group activities, peer consultation, and context-based language activities. Statistical analysis established a significant improvement in students’ pragmatic competence subsequent to the intervention (Mean = 34.63, SD = 8.41) compared to pre-intervention ratings (Mean = 24.27, SD = 7.11), with a large effect size (Cohen’s d = 1.99). Further statistical analysis investigated correlations between students’ communicative strategies and attitudes and demographics (grade and gender). Teachers’ ratings provided additional insights regarding perceived effectiveness and difficulties in using LCL. Implications include the advantage to incorporating learner-centered pedagogies to enable pragmatic competency gain in EFL settings and the value in offering recurrent teacher professional development. These findings suggest that classroom application of LCL approaches can meaningfully contribute to English pragmatic gain among Kurdish speakers.

## 1. Introduction

### 1.1 Background and context

The English language is a foreign language that has gained much notoriety, of course to the charge of the Iraqi English language in the Kurdistan region of Iraq. It features in School curricula as a compulsory subject, an introduction to global awareness, and has emerged as a wonderful medium of international communications. The region faces some odd but serious issues, such as a shortage of educational facilities, old-school and rote learning experiences, and nearly no real-world exposure to English. Rather than showing any gains towards achieving fluency and communicative competence in English, systemic problems hold students back [1]. Moreover, Kurdish regions suffer from learning English as a second language due to low socio-economic – school quality differences that evolve exponentially from countryside to city [2]. Communicative competence: pragmatic competence (knowing which characteristics are appropriate to use in particular social and cultural contexts), and semantic competence (knowledge of the pragmatic vocabulary—the politeness expressions and reporting devices—that a community draws upon to carry out these tasks). It is of critical importance, therefore, to document and describe this aspect of such learner talk so that learners can make sense of and produce the kinds of language that might conform to cultural and contextual norms among native and international interlocutors alike. This means the necessity of pragmatic competence in EFL learning. In a globalized world where English is the main language of international business, the language of diplomacy, and of higher education [3,4] , being an EFL learner with no pragmatic competence will not help Kurdish EFL learners be successful. You need pragmatic skills as well. For instance, students were able to “speak”, but still failed to transfer the language to the real-world context.

Barriers to acquiring pragmatic competence by Kurdish EFL learners. The biggest problem is the lack of real English use, which means there are no chances and opportunities to communicate with native or fluent English speakers. In Kurdish schools, grammar-oriented instruction denominates to a larger extent disregard its pragmatic features, such as strategic turn-taking, politeness strategies, and speech acts. Highly relevant cultural aspect is are another barrier. This makes learners become overwhelmed while studying English speak cultures as a social phenomenon due to this lack of communication in Kurdish culture. On the other hand, in Kurdish Regions, teachers are never taught how to teach skills in pragmatic instruction in their lessons, and therefore, students never learn these skills ( [5,6]).

### 1.2 Research gap

#### 1.2.1 Scope of pragmatic competence in Kurdish EFL learners.

While there has been recognition of the significance of pragmatic competence in English as a foreign language (EFL) learning in second language acquisition (SLA), the studies on Kurdish EFL learners are marginal. All of this makes issues relating to pragmatic competence rare phenomena within an Asian or European context so far, and offers a take on the specific sociolinguistic and cultural setting these Kurdish regions offer. Even more importantly, studies on problems of education in Kurdistan- Kurdistan Region of Iraq do not address the need for the most crucial aspect of communicative competence, which is the pragmatic competence necessary for active communication. The pragmatic development and use of interpersonal pragmatic skills by early Kurdish learners and how cultural and contextual issues shape them [7,8] are not included in this line of thinking. This gap is committed to bridging the gap so that the pedagogy can be tailored to the needs of Kurdish EFL learners.

#### 1.2.2 Exploring learner-centered in this context.

One more significant research gap on the learner-centered approach to teaching pragmatic competence is a less maximized area in the Kurdish EFL context. Kurdish schools are based on English teachers, rote learning, and grammar methodologies. But they cannot teach students the skills necessary to use their language in authentic, real-life situations. In keeping with what has been found in other EFL contexts, instructional interventions that have been found to be effective in developing pragmatic competence have involved active learner-centered approaches that emphasize active participation in learning at least in part, interactional processes in learning, and contextualized learning. There have been few studies regarding how such approaches were implemented and their impact in Kurdish classrooms. Since the learn-centered approaches are more beneficial for conducting effective pedagogical practices, it would deserve further research on the implementation of learn-centered methodologies in the context, with due sensitivity to cultural characteristics and resource constraints [9,10].

### 1.3 Objectives

1. To assess the impact of a learner-centered approach on pragmatic competence development.
2. To help incorporate the pragmatics-concerned teaching in Kurdish EFL classes.

### 1.4 Research questions

1. How does a learner-centered approach benefit the pragmatic competence of Kurdish EFL learners?
2. What are the obstacles Kurdish EFL learners face in accepting a learner-centered approach to pragmatics development?

## 2. Literature review

### 2.1 Pragmatic competence in EFL learning

Pragmatic competence is the ability of learners to use language in social and cultural contexts beyond the grammatical aspect to include politeness, speech acts, and conversational norms (Roever and [11]). It is especially challenging for EFL learners to accomplish pragmatic competence because what happens in the classroom involves form over function, and they don’t get ample opportunities to practice real communication [12,13]. Again and again, research has shown that students who possess a high pragmatic ability are able to negotiate meaning better, avoid misunderstandings, and participate in effective social and academic interaction.

### 2.2 Learner-centered approaches and pragmatics

Student-centric instruction has become popular in EFL settings because of its potential benefits in terms of language performance and communicative ability [14]. Methods focusing on cooperation, autonomy, and naturalistic interaction give learners a chance to exercise pragmatic skills in authentic situations [15]. Role-playing, peer conversations, and task-based exercises help students mimic practical communication in everyday life and exercise pragmatic skills. Current research has reaffirmed the findings that such methods contribute not only to linguistic achievements but even pragmatic sensitivity [16,17].

### 2.3 Studies in Kurdish and regional contexts

Few studies on pragmatic competence exist in the Kurdish EFL setting [18]. Explored Kurdish learners’ advice speech act production and found pragmatic inadequacies, especially in the application of proper politeness behaviors. Likewise [19], elaborated on the use of technology to promote pragmatic training in Northern Cyprus but established that Kurdish learners continue to lack practical application of pragmatic knowledge in natural discourse. Such findings are congruent with [13], who established that Turkish learners encountered speech acts and discourse implicatures difficulties, indicating a need for increased pragmatic development support among learners in the wider region.

#### 2.3.1 Literature gap: Kurdish learners related research.

Although there has been rising awareness of pragmatics in SLA literature, pragmatic competence among Kurdish EFL learners has not garnered enough attention. Preceding studies (S. H [18]) only concentrate on particular speech acts and do not systematically assess the effect of a learner-centered teaching methodology on pragmatic development. In addition, although international research in particular identifies the advantages of supporting learner autonomy and group work [14,15], there has not been much evidence of the effect of such methodology on pragmatic achievements in Kurdish university settings. Redressing the balance, the current study sets out to examine the effect of a learner-centered learning (LCL) methodology in promoting Kurdish EFL learners’ pragmatic competence at various academic levels.

## 3. Methodology

### 3.1 Research design

The quasi-experimental design was used in the study instead of a mixed-method or full experimental design owing to practical and ethical constraints in an academic setting. Random assignment was not possible as students were already enrolled in respective courses in university; therefore, intact classroom groups were used to maintain the natural learning context. Intervention was for six weeks and involved instructional materials such as authentic dialogue, scripted role-play, and situational exercises with emphasis on culture. Pragmatic features such as speech acts (requests, refusals, apologies), politeness strategies, and conversational implicatures were instructed explicitly through explicit instruction, directed discussions, and interactive practice. Role-play exercises simulated actual communication contexts, while peer interaction and group activities motivated students to apply pragmatics in context. Structured debates and problem-solving exercises were applied in simulations to reinforce language use in professionally and socially relevant contexts. Scaffolding techniques such as teacher modeling and corrective feedback ensured progressive learning to enable students to internalize and apply pragmatics optimally.

### 3.2 Participants

The present study investigates the views of the teachers and students of English as a Foreign Language (EFL) on the Learner-Centered Learning (LCL) approach and its influence on pragmatic competence acquisition. This study was conducted in the Kurdistan Region of Iraq by two different universities in Northern Iraq, where the education system functions with systematic admission policies formulated by the Ministry of Higher Education. Data collection at different times took place as follows: first-grade students (n = 38) were tested between April 1 and May 13, 2024, and fourth-grade students (n = 60) were tested between September 10 and October 22, 2023 and the number of participants was 98 students in total. This study engaged 12 teachers of English as a Foreign Language and 2 academic managers, as well as first- and fourth-grade students who took part in the instruction based on the LCL approach.

### 3.3 Intervention

A learner-centered status intervention characterized by active student involvement, student choice, and relevance to the student was utilized. It integrates these theoretical constructs and employs the synthetic approach to develop transactional and pragmatist learning theories that call on students to construct knowledge in their quest for interaction, reflection, and experience. It also involves an approach from active participation, task-based learning, scaffolded learning, cultural sensitivity, and feedback-driven learning. The activities aimed at developing learners in using English in social and professional contexts consist of role-playing of functions of speech, analysis of real dialogues, peer teaching or collaboration, and simulation of different real events. There are scenarios based that assess how well you can recognize and produce language in different scenarios. This range of activities is so inclusive that it includes theoretical understanding and practical ability in how to use English in various situations.

### 3.4 Data collection

The research employed a pre-post test design and student and teacher questionnaires to determine the impact of a learner-centered approach on Kurdish EFL learners’ pragmatic competence. A Discourse Completion Test (DCT) was employed as a primary instrument to measure pragmatic competence before and after treatment. It was a 10 real communicative scenario test to examine students’ capacity to perform effective speech acts of request, refusal, and apology by using English. The responses were rated for accuracy, appropriateness, and politeness strategy employment.

Besides that, two questionnaires with a structured format were given to students and teachers. Student questionnaires addressed learner-centered learning mindsets, participation in communicative activities, and difficulties in acquiring pragmatic competence. Teacher questionnaires explored instructors’ conceptions about learner-centered pedagogy, whether it is effective or not, and difficulties in implementation. Data collected provided quantitative information about students’ progress and whether it is possible to implement pragmatic instruction in Kurdish EFL classrooms.

#### 3.4.1 Ethical considerations.

This research used human participants and followed ethical research guidelines. Written informed consent was received from all participants before questionnaires and test material were given. Consent statements appeared in clear writing at the start of all paper-based measures, and participation was optional.

Information was gathered from two cohorts of Kurdish University students in the Kurdistan Region of Iraq:

- First-graders were assessed from April 1 to May 13, 2024.
- Fourth-graders were tested from September 10 to October 22, 2024.

All of those who took part were more than 18 years old during data collection. Institutional authorities gave ethical clearance, and the research was structured in such a way that it posed neither harm nor risk to participants.

### 3.5 Data analysis

In this research, Descriptive and inferential statistical tests were employed to determine whether or not the intervention was effective.

**Analysis of pretest and posttest scores.** A paired t-test was employed to compare post-test and pre-test scores to evaluate improvement in pragmatic competence. The Shapiro-Wilk test was employed to test normality assumptions, and Levene’s test to test homogeneity of variances. Effect size was estimated, and practical significance was reported using Cohen’s d.

Analysis of Questionnaire Data. Descriptive statistical methods (mean, standard deviation, frequency distribution) were applied to student and teacher Likert-scale responses to identify trends in learner-centered learning attitudes. An independent samples t-test was also employed to test for differences in perception between different subgroups (such as female and male students and first and fourth-year students).

#### 3.5.1 Quantitative analysis: statistical comparison of pre-and post-test scores.

**Descriptive statistics.** Results Descriptive analysis revealed a pre-test means score of 24.27 (SD = 7.11) and a post-test mean score on score 34.63 (SD = 8.41). The guiding mean of 10.36 indicates a learner-centered approach, which is in favour of pragmatic competence having been improved. The diversity of participants as well as the learning gains were indicated by the range of pre-test scores (10–39) and post-test scores (13–48).

**Fig 1:**
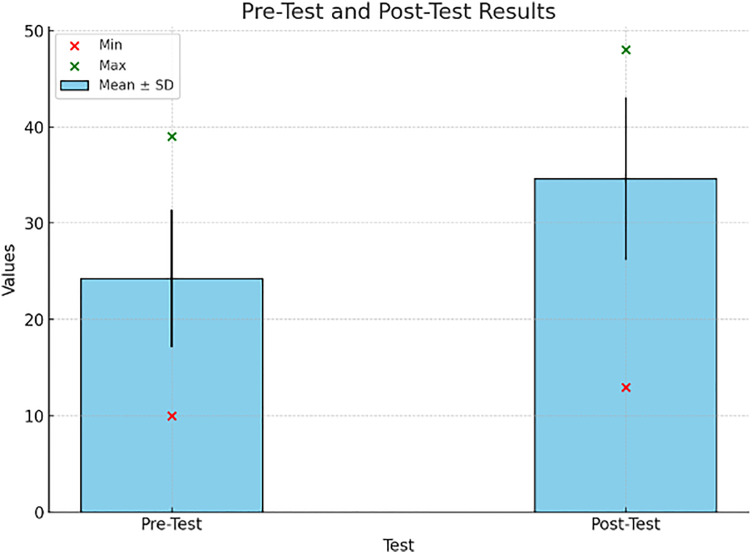
Pre- and post-test scores.

The first part of the descriptive analysis showed the mean pre-test score to be 24.27(SD = 7.11) and the mean post-test score 34.63(SD 8.41), which was statistically significant. It reflected a 10.36-point increase2, indicating the potential of Learner centered approach to assist in developing pragmatic competence (see Table 1 and Fig 1).

**Table 1:** Comparison of Pre- Test and Post-Test scores.

Test	Mean (M)	Standard Deviation (SD)	Minimum (Min)	Maximum (Max)
Pre-Test	24.27	7.11	10	39
Post-Test	34.63	8.41	13	48

**Fig 2:**
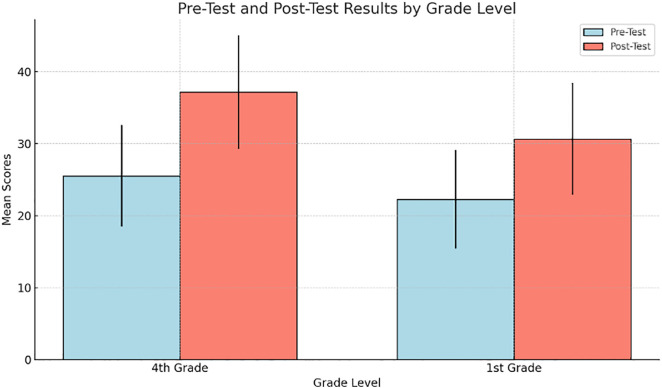
Pre-test and post-test scores by grade.

When results were analyzed by grade level, there was a significant improvement in 4^th^ year students than 1^st^ year students. T test confirmed a significant difference (p < 0.001), this showed that advanced learners benefited from this intervention (see Fig 2 and Table 2).

**Table 2:** Pre-Test and Post-Test Scores by Grade.

Test	Mean (M)	Standard Deviation (SD)	Sample Size (n)
Pre-Test	25.53	7.05	58
	22.26	6.82	40
Post-Test	37.15	7.86	58
	30.66	7.76	40

**Fig 3:**
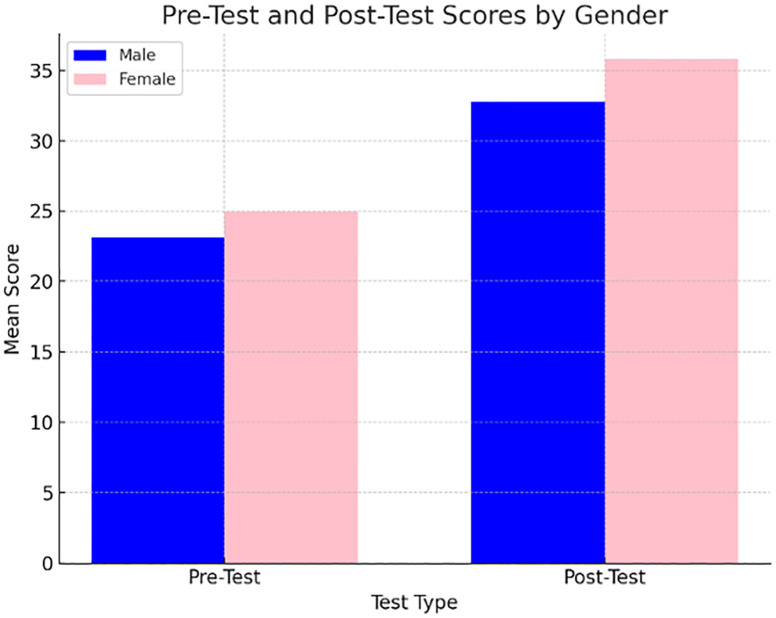
Pre-Test and Post-Test scores by gender.

After the analysis by gender was done, there was no observed difference between both genders. Here, p = 0.084, which showed that both gender benefited equally from the Learner-centered approach (see Fig 3 and Table 3).

**Table 3:** Pre-Test and Post-Test Scores by Gender.

Test	Gender	Mean (M)	Standard Deviation (SD)	Sample Size (n)
Pre-Test	Male	23.16	6.84	42
	Female	24.97	7.25	56
Post-Test	Male	32.79	8.27	42
	Female	35.80	8.35	56

**Fig 4:**
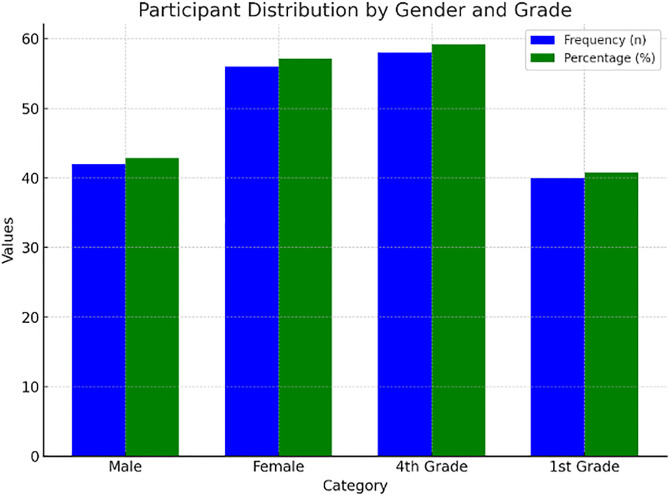
Participant distribution by gender and grade.

The participants distribution by gender and grade is illustrated in Fig 4 and Table 4.

**Table 4:** Participant Distribution by Gender and Grade.

Variable	Category	Frequency (n)	Percentage (%)
Gender	Male	38	38.78
	Female	60	61.22
Grade	4^th^	58	59.18
	1^st^	40	40.82

## 4. Results

### 4.1 Findings

The research explored whether and how a learner-centered methodology affects Kurdish EFL learners’ pragmatic competence and difficulties in adjusting to this pedagogy.

#### 4.1.1 Improvement in pragmatic competence.

The paired t-test showed that there was a significant improvement in students’ pragmatic ability following the six-week treatment. Pretest mean was 24.27 (SD = 7.11), and posttest was 34.63 (SD = 8.41), with a gain of 10.36 points (p < 0.001). This statistically significant improvement shows that learner-centered pedagogy enhances learners’ ability to identify and produce target speech acts such as requests, refusals, and apologies (see Table 5).

**Table 5:** Pre-test and Post-Test score.

Test	Mean(M)	Standard Deviation(SD)	Minimum(Min)	Maximum(Max)
Pre – Test	24.27	7.11	10	39
Post – Test	34.63	8.41	13	48

Subsequent breakdown by grade level indicated that fourth-grade students outperformed first-grade students with a post-test mean of 37.15 (SD = 7.86) compared to first-grade students with a mean of 30.66 (SD = 7.76) (p < 0.001), suggesting that more language proficient students better integrate pragmatic competences (see Table 6)

**Table 6:** Pre-Test and Post-Test Scores by Grade.

Grade Level	Pre – test Mean (M)	Pre –test SD	Post Test Mean (M)	Post Test SD
4^th^	25.53	7.05	37.15	7.86
1^st^	22.26	6.82	30.66	7.76

The statistical analysis (t-test) confirmed that there was a significant difference (p < 0.001), which indicated that more advanced learners benefited from this intervention.

Gender comparison showed that both males and females benefited equally by the intervention, as there was no difference found between post-test scores (p = 0.084) (see Table 7).

**Table 7:** Pre-Test and Post-Test Scores by Gender.

Gender	Pre-Test Mean (M)	Pre-Test SD	Post-test Mean (M)	Post – Test SD
Male	23.16	6.84	32.79	8.27
Female	24.97	7.25	35.80	8.35

Here, there was no significant difference observed between male and female learners in post-test performance (p = 0.084)

#### 4.1.2 Hindrances to adopting a lear.

Student survey responses indicated that restricted exposure to authentic English use (M = 4.21, SD = 0.87) and teacher-centered classroom norms (M = 4.03, SD = 0.92) were the biggest obstacles to learner-centered learning. Teachers also reported similar obstacles with limited training in pragmatics pedagogy (M = 4.17, SD = 0.84) and large class sizes (M = 3.95, SD = 0.89), having negative effects on implementation.

### 4.2 Summary of results

Most of the activity types that correlated with the improvement of students’ pragmatic competence were learner-centered activities. With such active and team learning, students gained basic knowledge of the language, including contextual communication, politeness strategy, and speech acts on certain occasions.

#### Roles of learner-centered activities.

The learner-centered approach was able to accord multiple advantages to facilitate the development of pragmatic competence through its interactive and task-based framework. Another thing that the previous approach had was one of the key features of simulating real-life scenarios. Roleplaying experiences gave students the ability to practice their pragmatic skills in authentic situations that often bridged the gap between what we were learning in the classroom and what was encountered in their day-to-day spoken discourse. The above activities also involved social interaction of an alternatively collaborative nature in which learners interact and participate, reconciling conflicting meanings, puzzles, and applying it in a variety of, especially human, contexts.

Moreover, the learning-centered approach helped the learner to become an autonomous and self-dependent learner. This approach breathed life into online students midway in their academics, enabling them to take charge of their study progress. The implication from this is that these students were then more comfortable using English pragmatically, which now highlights the significance of the approach to language development. The results evidence the need for learner-centered activities to foster students´ pragmatic competence. While learning a new language, they not only improve their command of the language but also develop the confidence and independence to create new ideas.

## 5. Discussion

### 5.1 Relating findings to existing research

The results concur with previous studies that identify learner-centered pedagogy for pragmatics competency development. As with Taguchi [20], whose study indicated that speech act recognition is enhanced by task-based learning among Japanese EFL learners, this study confirms that interactive tasks such as role-plays, peer activities, and simulations improve pragmatics usage.

Furthermore, this is consistent with DeKeyser [21], who argues that higher linguistic proficiency facilitates pragmatic awareness. This would mean that learner-centered pedagogy is beneficial for novices to learn from, yet there is a requirement for greater scaffolding to promote optimal development of pragmatic ability.

But student and teacher difficulties are indicative of systemic barriers to learner-centered practices in Kurdish EFL contexts. Basturkmen [22] encountered similar resistance in teacher-centered classrooms with traditional grammar-based pedagogy that represses communicative language acquisition. Teacher lack of training in pragmatics reported in this study is also supported by findings by Kasper and Rose [4], which demand teacher development programs to prepare instructors to integrate pragmatics instruction with ease.

### 5.2 Practical relevance to Kurdish universities

As a reaction to these issues and in order to facilitate more efficient pragmatic teaching, Kurdish universities can adopt the following reforms:

1 Integrating Pragmatics into EFL Curriculum

Current EFL syllabi should include explicit pragmatics teaching through learner-centered activities such as role-plays, authentic dialogue, and case studies. Pragmatic testing (e.g., discourse completion tasks) needs to be part of formal examinations in order to examine students’ linguistic capacity to use language in social contexts.

2 Teacher Training and Development

Universities should offer professional development courses on pragmatics pedagogy and learner-centered practices. Offering training sessions on speech act theory, politeness mechanisms, and discourse analysis would enable instructors to incorporate these ideas into their classes.

3 Increased Exposure to Real English

Virtual language exchange programs and partnerships with native English speakers can provide students with authentic communicative practice. Enabling the use of multimedia resources such as interactive simulations, role-play programs, and web-based forums can also foster pragmatic competence development.

4 Transcending Classroom Boundaries

Universities should consider reducing class sizes or employing small-group work in large classes to promote active engagement. Offering supplemental digital materials (such as online forums, interactive language software) can enable students to practice pragmatics outside of class.

### 5.3 Comparing it to other research

The results of this study are consistent with the trends of learner-centered EFL approaches as presented in the international literature. This demonstrates that role plays and real-world simulations are potent means for developing pragmatic competence (see [20], for a study of Japanese EFL learners). DeKeyser [21] also demonstrated that task-based learning generates meaningful communication. In the case of the Kurdish context, this study generalizes these findings into the dominating cultural and systemic difficulties that converge in the experience of Kurdish learning. As House’s [23] study points out, approaches that focus on the learner are also essential to negotiating complex social contexts. However, it also continues the discourse by examining how Kurdish learners with these different sociolinguistic barriers.

However, given that most studies may only consider linguistic competence, thus, this study combines culture, pedagogy, and acquiring pragmatic skills. It recognizes real issues with implementation, including large class sizes and resource limitations, and provides practical recommendations for adapting learner-centered solutions in resource-constrained contexts. A valuable addition to the growing body of literature that can be (and should be) under a more nuanced approach to backing up a learner-centered EFL pedagogy. These implications confirm the wider relevance of learner-centered strategies and encourage customized approaches to underrepresented settings such as Kurdish EFL classrooms. The current study is a practical and significant advance in this realm, as it provides valuable insight into more inclusive and effective language pedagogical practices.

### 5.4 Implications for EFL teaching in Kurdish contexts

The article helps to suggest the use of pragmatic-focused, learner-centered activities in Kurdish EFL classrooms. It refers to the use of role-plays and simulations using authentic materials, peer collaboration, explicit instruction on pragmatic points, reflective feedback, and task-based learning. Content is also customized for basic, guided activities for beginners within the app, and advanced activities for experts using the app. Cultural sensitivity, for instance, should come along with comparisons of politeness strategies in English and Kurdish. Use materials and mobile technology to address resource limitations. They can scaffold, though, in mixed ability classes. Therefore, with all these steps in mind, implementing learner-centered, pragmatic-focused approaches in foreign language teaching in Kurdish classrooms could support students to communicate in ‘real-world situations with confidence and cultural sensitivity.

### 5.5 Challenges of limitations

Kurdish EFL learners face unique challenges because of the differing communication styles of Kurdish and English cultures. These differences should be the focus of lessons to help address and raise awareness of them. With apps that learn your language and virtual exchanges with native speakers, your line on the dotted line a limited exposure to real-life spoken English. A smoother process can be created by using low to no-cost, most creative engagements (role-play scripts, peer-led activities) to work around resource limitations. Teachers require new instructional skills and a mindset change in order to adopt learner-centered pedagogy. Professional development programs focusing on learner-centered strategies enable teachers to gain new skills. Gradual implementation can ease resistance to change. Differentiated instructional methods with collaborative learning strategies address the needs of students with varying abilities in diverse classrooms. Adopting learner-centered pedagogy in Kurdish classrooms faces challenges, which are being addressed by supplying essential training and resources that allow teachers to implement this approach, leading to better pragmatic competence and dynamic learning environments.

## 6. Conclusion

### 6.1 Summary of findings

The learner-centered pedagogy enhanced Kurdish EFL learners’ pragmatics significantly with a post-test score improvement of 10.36 points (p < 0.001). The more proficient learners benefited more than beginners did, supporting the role of linguistic capacity in pragmatic development. The key implementation challenges are teacher-centered traditions, restricted exposure to authentic English, and a lack of teacher training in pragmatics. Pragmatic reforms, including curriculum changes, teacher training, and increased exposure to authentic communication contexts, are required to sustain pragmatic competence development in Kurdish EFL classes.

### 6.2 Contributions to EFL research

Kurdish contexts have been largely neglected in studies of pragmatic competence; consequently, this study seeks to help fill this gap by focusing on pragmatic skills such as speech acts, politeness strategies, and contextual language use. Focusing on the sociocultural and systemic challenges that Kurdish learners are faced, such as teacher-centered pedagogy and the lack of opportunities for real English usage. Furthermore, the study enriches the understanding of learners as to how to participate in the EFL learner-centered pedagogy, autonomously, and applied to real-world contexts. This study provides an example of how pedagogy for diverse proficiency levels and cultures might be reoriented to learner-centeredness, and it outlines implications for adapting this pedagogy to and across various EFL contexts.

### 6.3 Future research directions

Further research is needed based on the research conducted to study the long-run effect of learner-centered designed environments on pragmatic competence. This will tell us what accounts for the areas of pragmatic reaction that endure and what implications this has for broader outcomes such as agency and global communicative competence. If the outcomes of this research are to be met, then this can provide a cross-comparative of the learner-centered pedagogies in different sociocultural contexts, e.g., rural African societies, Southeast Asia classrooms, and indigenous language learners. Furthermore, the study would reveal general principles of learner-centered teaching, but also an exact adaptation to different contexts would prove to generate higher educational effectiveness. It would enable contextual appropriate strategies for developing communicative competence in EFL education in other parts of the world.
